# Immune Checkpoint Blockade in Gynecologic Cancers: State of Affairs

**DOI:** 10.3390/cancers12113301

**Published:** 2020-11-09

**Authors:** Maureen L. Drakes, Cheryl M. Czerlanis, Patrick J. Stiff

**Affiliations:** Department of Medicine, Cardinal Bernardin Cancer Center, Loyola University Chicago, Building 112, 2160 South First Avenue, Maywood, IL 60153, USA; cczerla@lumc.edu (C.M.C.); pstiff@lumc.edu (P.J.S.)

**Keywords:** endometrial cancer, ovarian cancer, cervical cancer, gynecologic cancers, immune checkpoint inhibitors, clinical trials, combination therapy, immune-related toxicity, disease improvement

## Abstract

**Simple Summary:**

Most endometrial cancer patients are diagnosed at an early stage, receive standard treatment, and survive well. Ovarian cancer has no specific symptoms and usually escapes diagnosis until the patient has advanced disease. This disease results in the highest number of deaths of gynecologic cancers. Current treatments for gynecologic cancers in the advanced stage are not sufficiently effective for good outcome in most patients. This review discusses two novel treatments, which are immune checkpoint inhibitor antibodies that block immune checkpoint molecules cytotoxic T lymphocyte associated protein-4 (CTLA-4) and programmed death-1 (PD-1) in patients. The antibody blocking of CTLA-4 or PD-1 alone is promising treatment for some categories of advanced disease endometrial cancer, but it has little effect against ovarian cancer. Our study primarily discusses the status of clinical trials for these two diseases and the biological parameters governing the different outcomes to these therapies. We also propose mechanisms whereby blocking CTLA-4 and PD-1 may be used in combination with other agents to give much better survival in advanced disease ovarian cancer patients.

**Abstract:**

This review provides an update on the current use of immune checkpoint inhibitors (ICI) in female gynecologic cancers, and it addresses the potential of these agents to provide therapy options for disease management and long-term remission in advanced disease patients, where surgery, chemotherapy, and/or radiation fail to meet this goal. The topic of immune checkpoint inhibitors (ICI) blocking cytotoxic T lymphocyte associated protein-4 (CTLA-4) and the programmed death-1 (PD-1) axis has come to the forefront of translational medicine over the last decade for several malignancies. The text will focus primarily on a discussion of ovarian cancer, which is the most frequent cause of death of gynecologic cancers; endometrial cancer, which is the most often diagnosed gynecologic cancer; and cervical cancer, which is the third most common female gynecologic malignancy, all of which unfavorably alter the lives of many women. We will address the critical factors that regulate the outcome of these cancer types to ICI therapy, the ongoing clinical trials in this area, as well as the adverse immune responses that impact the outcome of patients given ICI regimens.

## 1. Introduction

Uterine corpus endometrial cancer (endometrial cancer, EC) is the most frequently occurring gynecologic cancer in the Western world. It is estimated that in the United States, there will be 65,620 new cases of this disease in 2020, and a projected 12,590 deaths [1]. EC is generally diagnosed at an early stage and is well managed with surgery, radiation, and/or chemotherapy. More than 80% of patients diagnosed with early stage EC achieve a 5-year overall survival rate [1]. However, EC patients with advanced/recurrent disease have a poor outcome, such that those with distant metastasis have a five-year survival rate of approximately 16% [1]. This latter category of EC patients responds poorly to current management treatments, and hence, the use of novel therapies such as immune checkpoint inhibitors (ICI) is often considered for these advanced/recurrent disease EC patients.

High-grade serous ovarian cancer (HGSOC) results in the highest number of deaths among gynecologic cancers [2]. This disease has no characteristic symptoms, and due to the vague nature of symptoms observed in patients, it is difficult to detect in the early stages, and hence diagnosis most often occurs in the advanced/metastatic stages. Unfortunately, the majority of patients diagnosed with HGSOC have poor response to conventional management therapies, and most of these do not reach a five-year survival landmark. Statistics predict that in the U.S., 21,750 new cases of ovarian cancer will be diagnosed in 2020, and 13,940 patients will die of the disease [1]. With the current dismal outcome, there is a crucial need for novel treatment options for HGSOC.

It is predicted that cervical cancer will have 13,800 newly diagnosed cases in 2020, and there will be 4290 deaths due to this malignancy [1]. The cause of cervical cancer may be due to persistent human papilloma virus (HPV) infection in approximately 90% of cases diagnosed [3]. Prevention and detection measures for uterine cervical cancer include (HPV) vaccination, Pap smears, and HPV testing. However, about 5% of women in North America are diagnosed with stage IV cervical cancer, and the five-year survival rate for these women is 9.2. to 21.6% [4,5]. Vaginal cancer is probably due to HPV infections in 70% of the cases, and vulvar cancer is probably due to this pathogen in 70% of cases [3]. These cancers cause a substantial number of deaths in women annually, and several ICI and other novel therapy clinical trials are ongoing in an effort to provide better treatment options to improve survival in these patients. PD-L1 was shown by several investigators to be highly expressed in cervical and vulva cancers [6,7], even though the correlations between PD-L1 and ICI therapy outcome are not yet understood.

Immune checkpoint synapses consist of several co-inhibitory molecules that are primarily responsible for limiting T-cell receptor signaling and abrogating immune responses. This strategic process set in place by the immune system is useful to halt immune responses in individuals after microbial infections are resolved, or in the development of self-tolerance to limit autoimmune disease [8]. However, in cancer, high levels of immune checkpoint molecules on immune cells or on tumor cells are often associated with exhausted T cells, which are incapable of developing aggressive anti-tumor responses, as well as with resistance to several classes of therapy [9,10,11].

For clinical practice, the first Food and Drug Administration (FDA)-approved monoclonal antibody targeting immune checkpoints was ipilimumab, which targets the cytotoxic T lymphocyte associated protein-4 (CTLA-4). This treatment was followed by FDA approval of antibodies blocking the programmed death-1 (PD-1)/PD-l ligand (PD-L1) axis, treatment which has revolutionized the therapy of many solid cancers [12]. Interestingly, the efficacy of these treatments in some gynecological cancers has fallen short of the desired response rates. This review will focus primarily on outlining disparities at the molecular and cellular level, which may influence the differing response rates of endometrial and ovarian cancer to ICI, and it will briefly discuss some clinical trials in progress for other female gynecologic cancers including cervical, vulvar, and vaginal cancer.

## 2. Immune Checkpoint Junctions in Cancer Immunotherapy

**Figure 1:**
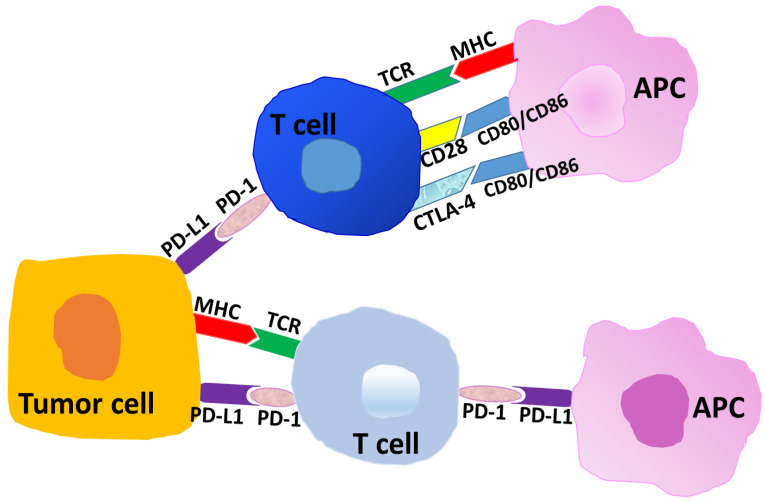
Representative schema of interactions at immune checkpoints. Tumor-associated antigens or neo-antigens are presented by the major histocompatibility complex (MHC) to T cells (T-cell receptor, TCR). The binding of CD28 on T cells to CD80/CD86 (B7-1/B7-2) on antigen-presenting cells (APC) results in heightened immune responses or anti-tumor immunity. The cross-linking of cytotoxic T lymphocyte associated protein-4 (CTLA-4) on T cells to CD80/CD86 on APC results in an inhibition of T cell responses. Programmed death-1 (PD-1) on T cells co-ligating with PD-L1 on APC or on tumor cells results in inhibitory or immune-suppressive responses in the tumor microenvironment.

Immune checkpoints (IC) present potent immune-suppressive mechanisms in cancer, and blocking of two of these pathways in particular has provided useful therapeutic alternatives to improve survival in many cancer types. Briefly, the binding of CD28 on T cells to B7-1/B7-2 (CD80/CD86) on antigen-presenting cells (APC) (Figure 1) results in co-stimulatory anti-tumor responses. However, co-inhibitory molecule CTLA-4 on T cells has a higher affinity for B7-1/B7-2 molecules than does CD28, and the preferential binding of CTLA-4 to B7-1/B7-2 blocks IL-2 release from T cells and limits T cell proliferation. In cancer, the development of an antibody to block the CTLA-4 to B7-1/B7-2 ligation leads to potent anti-tumor responses [13,14,15,16]. The first such CTLA-4 blocking antibody, ipilimumab, was FDA approved for metastatic melanoma in 2011 [12,17,18].

Immune checkpoint molecule PD-1 (CD279) is primarily expressed on T cells, and PD-L1 (CD274) is primarily expressed on antigen-presenting cells (APC), immunosuppressive macrophages, and tumor cells (Figure 1). These molecules generally show higher density in tumors, and the cross-linking of PD-1 to PD-L1 is a critical immune-suppressive component in the tumor microenvironment (TME). In cancers, including endometrial and ovarian cancer, the linkage of PD-1 to PD-L1 parallels with an environment rich in CD4+ CD25 high FoxP3+ T regulatory cells (T regs), high myeloid-derived suppressor cell (MDSC) activity, low cytotoxic T cell potential, and many other immunosuppressive parameters that trend to tumor progression [9,19,20,21].

In 2014, the FDA approved a monoclonal antibody pembrolizumab, blocking PD-1, for use in patients with metastatic melanoma. Pembrolizumab is now approved for several other cancer types including non-small cell squamous cell carcinoma, recurrent head and neck squamous cell cancer, and solid cancers with high microsatellite instability (MSI-H) or mismatch repair (MMR) gene defects, including endometrial cancer [22,23]. There are now several other FDA-approved antibodies targeting the PD-1 axis [12,24], many of which are in single and combination therapy clinical trials for ovarian cancer, endometrial cancer, and other malignancies [25,26].

## 3. Classification of Endometrial Cancer

Cancer of the endometrium is the most common gynecologic malignancy in the United States (Table 1), and it comprises approximately 7% of new cancers in women [1]. Endometrial carcinomas (EC) are a collection of distinct histologic subtypes. Traditionally, endometrial cancers have been classified into two Bokhman histopathologic categories, based on pathologic features, endocrine and metabolic factors, and prognosis [27]. Type 1 endometrial neoplasms represent International Federation of Gynecology and Obstetrics (FIGO) grade 1 and 2 endometrial cancers and constitute about 80% of endometrial cancers. These tumors are generally hormonally mediated and sensitive to estrogen, associated with obesity, and may be preceded by a precursor lesion such as endometrial intraepithelial neoplasia. Type 2 endometrial cancers account for 10 to 20% of endometrial cancers and include FIGO grade 3 endometrial cancers and non-endometrioid, clinically aggressive histologies such as clear cell, serous, mixed cell, and undifferentiated. These tumors lack estrogen sensitivity, are not associated with obesity, tend to be high grade tumors, and are associated with diagnosis at a later stage and poorer prognosis. Tumors in the type 2 subgroup are associated with a higher rate of p53 mutations and overexpression of HER2/neu [28,29].

Recently, the Cancer Genome Atlas analysis stratified endometrial cancer (EC) into four distinct molecular subtypes as follows: polymerase ε (POLE)-mutant ultramutated, microsatellite instability high (MSI-H, hypermutated), copy number low, and copy number high [30]. The hypermutated group generally carries a high number of mismatch repair (MMR) defects [30,31]. The usefulness of these molecular classifications to correlations of patient outcome to ICI therapy will be addressed in subsequent sections.

In EC, survival is primarily controlled by disease stage at the time of diagnosis, histologic subtype, and tumor grade. Generally, in this disease, the majority of women present with uterine bleeding and are diagnosed at an early stage. Early stage disease is highly amenable to treatment with surgical resection, followed by adjuvant therapy with radiation and/or cytotoxic chemotherapy based on clinico-pathologic factors such as disease stage, histology of the tumor, grade, and tumor size. About 67% of endometrial cancers are diagnosed with disease confined to the uterus, resulting in a high survival rate of 95% at five years [1,32]. However, prognosis is significantly worse for patients diagnosed with regional or distant metastasis, with 69% and 16% five-year survival, respectively [1]. Patients with metastatic and/or recurrent EC often have low response rates to chemotherapy, and they have essentially run out of effective therapy management options. Therefore, this underscores the need to develop novel therapeutic approaches such as the administration of ICI for patients with advanced disease EC.

## 4. Cellular and Molecular Regulation of Endometrial Cancer Prognosis

Similar to most cancer types, the tumor microenvironment (TME) of EC consists of immunosuppressive lymphoid and myeloid cells, soluble molecules, and other pro-tumor elements that may limit patient success to novel and conventional therapies [33,34]. In a study of endometroid adenocarcinoma (EA) patients, investigators studied the relationship of inflammatory immune cells including lymphocytes, macrophages, and dendritic cells with disease outcome. These investigators evaluated archived histological material of 82 patients with stage I to III EA, with good (survival) and poor (disease progression and death) outcome. Outcome status was retrospectively determined from their patient study database [35]. All cases were stained with antibodies to identify CD3 (T cells), CD20 (B cells), CD57 (NK cells), CD68 (macrophages), and S100 (dendritic cells) by immunohistochemistry. Expressions of CD3, CD57, and CD68 were significantly higher in archived tissue in the good outcome group (*p* < 0.001) compared with the poor outcome group, whereas there was no significant difference between CD20 and S100 in the two groups [35]. High levels of immune cells, notably CD3 T cells in cancer tissue, and good outcome is consistent with the findings of other investigators [36,37,38].

However, in the case of EC, there are overriding considerations that shape the outcome of responses to ICI, and the subsequent text will focus on these parameters. The Cancer Genome Atlas classification (2013) of EC is particularly useful for the prediction of disease prognosis. As earlier mentioned, these molecular groupings are polymerase ε (POLE)-mutant ultramutated, microsatellite instability high (MSI-H, hypermutated), copy number low, and copy number high [30]. The MSI-H hypermutated group carries a high number of MMR defects and is most easily regulated by immunotherapeutic agents [39,40].The function of the MMR pathway is to repair single-strand breaks, mispairings, as well as small insertions or deletions that occur during DNA replication. Germline MMR deficiencies of one of four DNA MMR genes (*MLH1, PMS2, MSH2*, or *MSH6*) are associated with Lynch syndrome [41], which affects between 2 and 6% of endometrial cancer patients [42,43]. Yet, most of the MMR pathways deficiencies are due to somatic mutations [44,45].

In tumors, a high CD3+ and CD8+ T cell density indicates an active immune response against cancer cells and correlates with better prognosis in several cancers, including endometrial cancer. POLE-ultramutated and MSI/MMR deficiency (MMRd) tumors generally have high CD3+ and high cytotoxic CD8+ T cells, correlating with the best outcome of the four EC groups [36,37,38]. Importantly, EC was found to have the highest prevalence of MSI of 30 tumor types. About 30% of primary EC are MSI-H, whereas 13% to 30% of recurrent EC are MSI-H or MMRd [39,46,47,48,49].

Tumor mutational burden (TMB) is the total amount of somatic (acquired) mutations in a tumor [50]. Highly mutated tumors generally have an abundance of tumor-specific mutant epitopes, which act as neoantigens and are recognized as non-self and provoking immune responses [50,51]. Immune checkpoint inhibitors have shown promising efficacy against hypermutated cancers such as melanomas, lung cancers, and EC [22,52,53]. These cancer types have more tumor-specific neoantigens that stimulate the recruitment of more immunocompetent tumor-infiltrating lymphocytes (TILs) to augment anti-tumor immunity. Tumors with higher neoantigen load are associated with improved overall survival and increased tumor cell cytotoxicity parameters, including the expression of T cell receptor (TCR), interferon-γ (IFN-γ), and tumor necrosis factor (TNF) receptor pathway genes [54].

One report showed that POLE-ultramutated and MSI-H EC tumors also have an overexpression of PD-L1 [46]. Interestingly, there is still great debate concerning the interpretation and relevance of PD-L1 expression on immune or tumor cells across several tumor types and its relationship to ICI efficacy in cancer [55]. However, it is believed that tumors such as EC, with elevated TIL numbers and high tumor mutational burden, are more easily recognized and targeted by T cells [39,46,47,56,57]. Such tumors typically respond well to ICI therapy [58], with patients showing significant disease improvement and improved overall survival (O/S), as is often the case for EC patients selected for ICI therapy.

## 5. Immune Checkpoint Blockade Therapy in Endometrial Cancer

Immune checkpoint inhibitors have shown efficacy in multiple advanced solid tumors, predominantly among MMRd and MSI-H cancers and those with a high tumor mutational burden, such as EC (Table 2). Some of these studies will be discussed in the subsequent text in relation to EC treatment.

An early signal of clinical activity of immune checkpoint inhibitors in advanced endometrial cancer was seen in a phase 2 study of 41 heavily pretreated patients with metastatic carcinoma with or without MMRd. Subjects were treated with pembrolizumab, which is a fully humanized immunoglobulin monoclonal antibody against PD-1. The treatment was associated with an immune-related objective response rate (ORR) of 71% and an immune-related progression-free survival (PFS) of 67% in MMRd non-colorectal cancers. A total of two patients with endometrial cancer were enrolled. One of these exhibited a complete response, and the other exhibited a partial response [59]. High somatic mutation burden was associated with prolonged PFS (*p* = 0.02) [59].

The phase II KEYNOTE-158 study evaluated the anti-tumor activity and safety of pembrolizumab in previously treated, advanced non-colorectal MSI-H/MMRd cancers [60]. Patients were treated with a fixed dose of pembrolizumab 200 mg IV once every three weeks for two years or until disease progression, unacceptable toxicity, or patient withdrawal. Among patients with a broad range of solid tumors including 27 tumor types, there were 49 patients with endometrial cancer (21% of the treatment population). In the cohort of patients with endometrial cancer, the ORR was 57.1%, with eight patients (16%) achieving a complete response and 20 patients (41%) achieving a partial response. The median PFS was 25.7 months. In the entire study cohort of 233 patients, 64.8% of patients had treatment-related adverse events and 14.6% had grade 3 to 5 treatment-related adverse events, with one grade 5 event related to pneumonia. The most common treatment-related adverse events were fatigue, pruritus, diarrhea, and asthenia. This study further indicated that MSI/MMRd status could be a predictor of the response to PD-1 blockade in endometrial cancer [60].

Pembrolizumab was subsequently approved by the FDA in 2017 for the treatment of MSI-H or MMRd solid tumors, regardless of tumor type, with progression following treatment and for which there are no satisfactory alternative treatment options [22]. In June of 2020, the FDA labeling was extended to include patients with unresectable or metastatic tumor mutational burden-high solid tumors (TMB-H; ≥10 mutations/megabase [mut/Mb]) after prior therapy and in the absence of other treatment options. Simultaneously, the FDA approved the FoundationOne^®^ CDx (Foundation Medicine) test as the companion diagnostic for pembrolizumab to identify patients with solid tumors that are TMB-H (≥10 mutations/megabase) (pembrolizumab FDA package insert, June 06/20; https://www.accessdata.fda.gov/drugsatfda_docs/label/2020/125514s071s090lbl.pdf).

Table 2 summarizes some immune checkpoint inhibitors (ICI) monotherapy trials blocking the PD-1 axis in endometrial cancer (EC) patients, evaluating the success of agents other than pembrolizumab. Overall, even though there is good outcome in some monotherapy trials blocking PD-1 or PD-L1 in EC (up to 57.1% ORR), a greater cohort of EC patients may benefit in combination therapy designs using ICI and other agents, which may potentially alleviate suppressor mechanisms in the tumor microenvironment (TME). Combination therapy has the potential to afford additive or synergistic benefits, as compared to single agent treatment, as well as to overcome resistance mechanisms that are observed with ICI monotherapy administration, due to the upregulation of alternative immune checkpoint molecules or to emerging resistance caused by the presence of cells such as myeloid-derived suppressor cells (MDSCs) [61,62,63,64,65]. Currently, several clinical trials are ongoing with ICI treatment in EC patients, which are used in combination with cytotoxic chemotherapy, other ICI, vaccines and other immunotherapies, or targeted therapies [25,26,29,32,66].

For example, immune checkpoint inhibitor treatment has also been combined with targeted therapy agents such as lenvatinib. Lenvatinib is an oral multikinase inhibitor of vascular endothelial growth factor receptor 1–3 (VEGFR1-3), fibroblast growth factor receptors (FGFR) 1–4, platelet-derived growth factor receptor (PDGFR) alpha, c-Kit, and RET proto-oncogene. Pre-clinical data suggest that this agent induces immune activation via decreasing tumor-associated macrophages, which may lead to an increase in CD8+ T cells and enhanced anti-tumor activity [72].

KEYNOTE-146/Study 111 was a single-arm, open label, phase Ib/II study to evaluate the safety and efficacy of lenvatinib plus pembrolizumab in advanced solid tumors, including endometrial carcinoma [73]. Patients received lenvatinib 20 mg once daily orally plus pembrolizumab 200 mg IV once every three weeks, based on the recommended dosing from the phase Ib portion of the study. The final primary efficacy analysis was reported for the patient cohort with advanced endometrial carcinoma. The primary endpoint was ORR at 24 weeks (ORR_Wk24_). The ORR_Wk24_ was 38% in the cohort of 108 patients who were previously treated with conventional therapy. For 94 patients with MSS/MMRp tumors, ORR as measured by immune-related RECIST (irRECIST) was 37.2% versus 63.6% for 11 patients with MSI-H/MMRd tumors [73].

The safety profile of lenvatinib plus pembrolizumab was generally similar to that previously reported for each drug alone with the exception that hypothyroidism was reported at higher rates than previously observed for either monotherapy. Grade 3/4 treatment-related adverse events were seen in 68% of patients, and 17.7% of patients discontinued one or both therapies because of treatment-related adverse events. Overall, 19 patients (15.3%) discontinued lenvatinib, 15 (12.1%) discontinued pembrolizumab, and 11 (8.9%) discontinued both study drugs [73]. Based on the outcome of these studies, lenvatinib in combination with pembrolizumab was granted accelerated approval by the FDA in September of 2019 for the treatment of patients with advanced endometrial carcinoma that is not MSI-H or MMRd, and who have disease progression following prior systemic therapy and are not candidates for curative surgery or radiation [73].

From the studies summarized in the preceding text, it is evident that blocking the PD-1 axis in EC patients with monotherapy treatment may result in an ORR as high as 57.1%. ICI therapy in combination with targeted therapy lenvatinib had a better outcome of 63.6%. Based on these and other studies, ICI treatment is generally regarded as very promising as an alternative therapy option for advanced/recurrent EC. A more detailed list of ongoing EC clinical trials using monotherapy and combination therapy regimens are summarized elsewhere [25,26,29,32].

## 6. Pathology and Classification of Ovarian Cancer

Epithelial ovarian cancer is the fifth leading cause of cancer deaths among U.S. women, and it accounts for approximately 5% of all cancer deaths in women. This disease leads to more deaths than any other cancer of the female reproductive system (Table 1) [1]. Epithelial carcinoma is the most common histologic type of cancer of the ovary, fallopian tube, and peritoneum, accounting for 90% of all cancers at these sites. Epithelial ovarian carcinoma encompasses a heterogeneous group of neoplasms with multiple histologic subtypes, the most prevalent of which is HGSOC, followed by endometrioid carcinoma, clear cell carcinoma, mucinous carcinoma, and low-grade serous carcinoma (LGSOC).

These histopathologic types are biologically distinct diseases, as indicated by differences in epidemiological and genetic risk factors, precursor lesions, patterns of spread, molecular pathogenesis, response to treatment, and prognosis [74]. The immunohistochemical profiles and molecular biology differ among the histologic subtypes. Approximately 96% of patients with this disease have TP53 mutations and many also have BRCA1/2 mutations [75,76], resulting in chromosomal instability. LGSOC patients often carry KRAS and BRAF mutations [77]. Advances in the understanding of the pathogenesis and molecular biology of ovarian carcinoma are essential to developing new treatment options for this disease. HGSOC is believed to arise from the ovarian-surface epithelium and/or the fallopian epithelium [78]. A notable feature of HGSOC is the development of ascites fluid in the peritoneum, which facilitates the adhesion of cancer cells to the omentum and the serous membranes of the peritoneal organs [79] and promotes the development of cancer lesions at these sites shortly after the primary disease is established [80,81].

As there is no effective screening method, a late stage at diagnosis is common (HGSOC) and largely accounts for the low survival in ovarian cancer. Surgical staging and cytoreduction, followed by adjuvant cytotoxic chemotherapy, constitute the predominant management strategy for most women with ovarian cancer. With these treatment measures, there is a five-year survival rate of greater than 80% for patients diagnosed with early-stage disease [82]. However, about 75% of patients have loco-regionally advanced or metastatic disease at the time of diagnosis [83]. Standard therapies do not effectively manage HGSOC, and most of these patients relapse and die from their disease within five years; thus, the need for novel innovative therapies for HGSOC is a critical need.

## 7. Parameters Influencing the Response of Ovarian Cancer to Immune Checkpoint Inhibitors

Worldwide, in 2018, approximately 295,414 cases of ovarian cancer were diagnosed, and about 184,799 deaths occurred due to this disease [84]. With these figures, there is a compelling need for novel treatments in HGSOC. Progress in ICI immunotherapy has been very encouraging for many cancers [12,23,52,53], but unfortunately, that has not been the case for ovarian cancer [85,86].

This may be due to several reasons. Notably, the ovarian cancer TME consists of a unique immunosuppressive network that limits the benefits of novel and conventional therapies. This TME harbors cancer cells, pro-tumor immune cells, and endothelial cells, which are meshed together with a myriad of receptor–ligand interactions and soluble molecules, many of which facilitate tumor cell proliferation, disease progression, and chemo-resistance.

Some immunosuppressive cells in the ovarian TME are tumor-associated macrophages (TAMS), which have an upregulation of genes associated with the extracellular matrix (ECM) [87,88,89,90]. Additionally, myeloid-derived suppressor cells (MDSCs) enhance stemness and promote metastasis, induce resistance to therapy, and limit the anti-tumor functions of T cells through MDSC signature molecules, nitric oxide (NO), and arginase-1 (Arg-1) [91,92]. Elevated MDSC numbers are highly associated with resistance to conventional and novel therapies [61,62,93].

Other negative (pro-tumor) regulators in cancers, including ovarian cancer, are M-2 macrophages [94], N-2 tumor-associated neutrophils (TANS) [95], immature dendritic cells (DCs) [96,97], plasmacytoid DCs [98], T regulatory cells (T regs), T cells expressing chemokine receptor CCR4, exhausted T cells expressing immune checkpoint molecules PD-1, CTLA-4, or lymphocyte activation gene-3 (LAG-3; CD223) [99,100,101], and natural killer (NK) cells with impaired functions [102].

Some of the prime molecules that foster pro-tumor immunity in the ovarian TME are vascular endothelial growth factor (VEGF), indoleamine-2,3-dioxygenase (IDO), transforming growth factor beta (TGF-β) and interleukin 10 (IL-10), which are secreted by cells such as immunosuppressive TAMS, CD4+ CD25high FoxP3+ Tregs, and pDCs [103].

Yet in a complex and intricate TME such as in ovarian cancer, there are also immune enhancing elements, including M1 macrophages [104], N1 TANS [95], Batf3 lineage CD103+ DCs [105,106], mature DCs [107], and immunocompetent NK cells [108]. Additionally, anti-tumor T cells (tumor-infiltrating lymphocytes; TILs) are critical to mounting immune responses in ovarian cancer. These TILs recognize cancer antigens or overexpressed self-antigens, which have been processed and presented by antigen-presenting cells and elicit immune responses to these antigens [109,110,111]. CD8+ T cells and CD3+ T cells liberate anti-tumor molecules such as interferon-γ (IFN-γ) and IL-2, and cytotoxic molecules perforin and granzyme B are also secreted by effector CD8+ T cells, making them effective to kill tumor cells. CD103 intraepithelial (TILS) are primarily located in the ovarian tumor epithelium and are associated with survival [112].

In addition to the foregoing parameters, many of which confer significant immune suppression in the ovarian TME and hamper the efficacy of ICI treatment [113], there are two prime considerations that also determine how HGSOC patients respond to these inhibitors. The first of these critical factors is the density of CD3+ and CD8+ T cells in the TME. Cancer types that consists of high numbers of these TILs are classified as hot tumors and are most likely to exhibit a good outcome with ICI treatment [114,115,116,117]. Generally, cancers such as melanoma and non-small cell lung cancer (NSCLC) are in this category. Cold tumors are those consisting of a low density of TILs [114,115,116,117], and tumors such as prostate, pancreatic, or neuroblastoma fall into this category [118]. Ovarian cancer has a moderate to low number of TILs, and such patients are prone to give only a modest immune response to ICI.

The second of these crucial parameters which is likely to influence the prognosis of ovarian cancer patients receiving ICI therapy is the TMB. Ovarian cancer (unlike most EC) is classified as low to moderate TMB [118]. With these two contraindicating factors, low to moderate TIL infiltration and/or low to moderate TMB as in ovarian cancer, it stands to reason that this cancer will have only a limited response to ICI and possibly to most immunotherapy, as is often observed in clinical trials. These concepts are important and need to be taken into consideration in the therapy design phase of effective combination treatment regimens using conventional and/or immunotherapeutic agents to render ovarian cancer responsive to ICI therapy.

## 8. Immune Checkpoint Inhibition Therapy in Ovarian Cancer

Several clinical studies have evaluated the role of immune checkpoint blockade monotherapy in ovarian cancer. Response rates for single-agent checkpoint inhibitors have been low in this setting. A phase II study evaluated the safety and antitumor activity of nivolumab at 1 or 3 mg/kg every two weeks in patients with platinum-resistant ovarian cancer [119]. The best overall response rate was 15%, which included two patients with a durable complete response. Grade 3 or 4 treatment-related adverse events were reported in 40% of patients. Response to nivolumab was not related to the level of PD-L1 expression [119].

The KEYNOTE-100 final analysis revealed that pembrolizumab in 376 patients with advanced, recurrent ovarian cancer, was associated with modest anti-tumor activity [120]. Patients with epithelial ovarian, fallopian tube, or primary peritoneal cancer, with recurrence following front-line platinum-based therapy, received pembrolizumab 200 mg IV every three weeks for two years or until progression, death, or unacceptable toxicity. The primary study endpoint was ORR by RECIST v1.1 and by PD-L1 expression using the combined positive score (CPS). Patients in cohort A (*n* = 285) were less heavily pre-treated and had a platinum-free or treatment-free interval (PFI/TFI) of ≥3 to 12 months, while patients in cohort B (*n* = 91) had received 3–5 prior lines of chemotherapy and had a PFI/TFI of ≥3 months. ORR was 8.1% and 9.9% in cohorts A and B, respectively. Higher PD-L1 expression (measured by CPS score) was associated with an increased ORR: 5% in patients with CPS < 1, 11.6% with CPS ≥ 1, and 18.2% with CPS ≥ 10. The safety profile was similar to pembrolizumab monotherapy studies in other tumors [120].

Another study investigated the safety and efficacy of avelumab for patients with recurrent or refractory ovarian cancer (JAVELIN Solid Tumor trial; NCT01772004), giving modest outcomes in the response range as discussed for other ovarian cancer clinical trials as above [86]. In an expansion cohort of the phase Ib, open-label JAVELIN study, 125 patients with ovarian cancer received avelumab 10 mg/kg IV every 2 weeks. The ORR was 9.6% (Table 3), with a median overall survival of 11.2 months, and grade 3 or 4 treatment-related adverse events occurring in only 7.2% of patients. Notably, the patient population was heavily pretreated, with a median of three prior lines of therapy [86].

Dual immune checkpoint blockade strategies in ovarian cancer have also been investigated. Trial NRG-GY003 evaluated ipilimumab plus nivolumab compared with nivolumab alone in women with persistent or recurrent epithelial ovarian cancer [122]. Subjects were randomized to intravenous nivolumab (every 2 weeks) or induction with nivolumab plus ipilimumab for 4 doses (every 3 weeks), which was followed by maintenance nivolumab every two weeks for a maximum of 42 doses. The primary endpoint was objective tumor response within 6 months of enrollment. Secondary endpoints included PFS and OS, which were stratified by last platinum-free interval. One hundred patients were assigned to receive either nivolumab (*n* = 49) or nivolumab plus ipilimumab (*n* = 51). In the six-month study period, there were six (12.2%) responses in the nivolumab group and 16 (31.4%) in the combination group (odds ratio, 3.28; 85% CI, 1.54 to infinity; *p* = 0.034). The median PFS was 2 and 3.9 months in the nivolumab and nivolumab plus ipilimumab groups, respectively. Grade ≥ 3 related adverse events occurred in 33% of patients in the nivolumab group and 49% occurred in the combination group, with no treatment-related deaths. The combination of nivolumab and ipilimumab, compared to nivolumab alone, led to an increased response rate (31.4% versus 12.2%) and a marginal increase in PFS (3.9 versus 2 months) [122].

Other ongoing trials include a phase II study of neoadjuvant chemotherapy plus durvalumab and tremelimumab in advanced stage ovarian cancer (KGOG3046; NCT03899610) [123] and an umbrella study of biomarker-driven targeted therapy in patients with platinum-resistant recurrent ovarian cancer. The latter, a Korean Gynecologic Oncology Group study (KGOG 3045; NCT03699449), includes an arm evaluating durvalumab, tremelimumab, and chemotherapy in patients with low PD-L1 expression.

The foregoing text shows that the outcome of reported studies using ICI in ovarian cancer have only resulted in modest efficacy. Therefore, rational immunotherapy combinations are needed to improve the response rates especially in cancers such as ovarian cancer, where ICI alone only results in modest outcome, which is possibly due to many avenues of immune resistance [124,125,126]. For example, agents such as chemotherapy and radiation used to reduce tumor burden can be used in combination with ICI, which can boost anti-tumor T cell responses. Inhibitors blocking MDSC function to alleviate TME immune suppression combined with ICI can amplify the re-invigoration of T cells, resulting in more efficient anti-tumor T cell responses and better prognosis. The use of anti-PD-1 blocking agents combined with novel ICI such as anti-LAG-3 antibody can limit emerging resistance due to the upregulation of alternate immune checkpoints, which may occur when PD-1 is abrogated with antibody blocking therapy. ICI can also be combined with DC vaccines, which can augment the generation of tumor antigen-specific effector CD8+ T cells, resulting in more effective anti-tumor T cell immunity and improved disease outcome. ICI therapy may also be used in combination with anti-angiogenic inhibitors (such as anti-VEGF antibodies) [127], poly (ADP ribose) polymerase (PARP) inhibitors [128], or epigenetic modifiers (such as decitabine) [129,130], all of which can result in heightened CD8+ T cell anti-tumor responses through a variety of mechanisms.

Some combination therapy clinical trials under investigation are as follows. Anti-VEGF therapy may enhance immunotherapeutic responses when combined with immune checkpoint inhibitors. Bevacizumab, an anti-VEGF angiogenic monoclonal antibody, was studied in combination with nivolumab (anti-PD-1 antibody) in a phase II study in recurrent ovarian cancer [127]. The combination of nivolumab and bevacizumab demonstrated clinical activity in women with recurrent ovarian cancer, with an overall confirmed response rate of 29% [127].

PARP proteins is a family of 17 enzymes involved in a number of cellular processes. PARP-1 and PARP-2 regulate DNA damage repair. Homologous repair (HR) of double-stranded DNA breaks is dependent on many proteins including BRCA1 and BRCA2. BRCA1/BRCA2 mutations are common in HGSOC (22% patients) [76], and these mutant tumors are especially sensitive to PARP inhibition. PARP inhibition is associated with direct cytotoxic effects as well as a possible mechanism for augmentation of anti-tumor immunity in combination with checkpoint inhibitors. The combination of PARP inhibition along with CTLA-4 blockade (tremelimumab) (NCT02571725 and NCT04034927) and PD-1 (NCT02657889, NCT03522246, NCT03740165, NCT03824704 and NCT03955471) and PD-L1 (NCT03642132) blockade is being investigated.

Other ovarian cancer clinical trials investigating ICI and in combination with other agents are summarized elsewhere [66,131,132,133]. The outcome of these trials and the modest success rate to ICI when used in monotherapy or in combination therapy for ovarian cancer indicates that there are yet many unknowns in the realm of ICI therapy. Effective combination agents should act in synergy with ICI to alleviate immune suppression in the ovarian TME to boost the recruitment of immunocompetent TILs to tumor beds, and/or to induce tumor-specific neo-antigens, with the ultimate goal of rendering patients responsive to ICI treatments and effectively managing HGSOC.

## 9. Cervical and Other Female Gynecologic Cancers

Surgery is the most frequently used treatment for early-stage cervical cancer, and it is often met with great success. Conventional treatment options for metastatic/recurrent cervical cancer additionally includes radiotherapy and chemotherapy, and this treatment is most often not sufficiently effective for disease management at this late stage. Global reports indicate that in 2018, there were about 569,000 cases of cervical cancer worldwide, with 311,000 deaths [134]; Cancer Fact Sheets: Cervical Cancer. http://gco.iarc.fr/today/data/pdf/factsheets/cancers/cancer-fact-sheets-16. (accessed on 20 July 2019). With these figures, it is evident that similar to advanced disease EC and ovarian cancer, there is the urgent need for novel treatment options such as ICI therapy to reduce the percentage of fatalities due to cervical cancer.

Significant immunosuppressive parameters have been described in cervical cancer including tumor-positive lymph nodes with low CD8+ T cell/T regs ratio [135,136]. Furthermore, several studies have reported that there is a very high expression of PD-L1 in this disease [137,138,139]. In patients, HPV positivity correlated with increased PD-L1 expression [140]. The distribution of PD-L1 is on the surface of cervical cancer cells, TILs, and APCs, with PD-1 on T cells in the stroma of cervical cancer. The PD-1/PD-L1 axis has been targeted in several clinical trials [25,134], some of which are noteworthy in this report.

Clinical trial NCT02054806 was a phase Ib study of a cohort of 24 patients, expressing PD-L1 on tumor or stromal cells, with advanced cervical squamous cell cancer, in which pembrolizumab was found to be well tolerated and having some clinical efficacy [141].

In a phase II KEYNOTE-158 trial, pembrolizumab was investigated in a single cohort trial of 98 patients with recurrent/metastatic cervical cancer. Of 77 patients, the ORR was 14.3% (95% CI: 7.4, 24.1), with 11.7% partial responses and 2.6% complete responses, whereas no responses were found in patients with tumors not expressing PD-L1 (NCT02628067) [60]. With this outcome, pembrolizumab was subsequently approved in 2018 for recurrent/metastatic cervical cancer patients with PD-L1 positive tumors.

CheckMate 358 was a phase I/II basket clinical trial investigating nivolumab for virus-associated tumors, of which a cohort of 18 patients had cervical cancer, 17 of whom also had prior treatment with radiotherapy. Nivolumab treatment was given for up to 2 years every two weeks. Encouragingly, the resultant ORR was 26.3% (5 patients), giving a median O/S of 21.9 months in the entire cohort.

Vulvar cancer is one of the less frequently diagnosed gynecological cancers, with 6120 new cases predicted to be encountered in the US in 2020 and 1350 deaths expected due to this disease (Table 1) [1]. PD-L1 is reported to be expressed in most vulva squamous cell carcinoma [6,142,143], even though the significance of this parameter is not yet well understood. Clinical trials for vulvar and vaginal cancer are often part of basket studies of HPV-associated cancers (CheckMate 358). A phase I clinical trial, NCT03277482, is ongoing investigating the safety and efficacy of durvalumab and tremelimumab along with radiation therapy for recurrent/metastatic gynecological cancers.

## 10. Immune Related Toxicity

Immune checkpoint inhibitors (ICI) are associated with a broad spectrum of unique immune-mediated toxicities, requiring expert management, as these toxicities may occasionally be life threatening [144]. Immune-mediated toxicities can affect most organ systems and are believed to arise from autoimmune inflammatory complications of ICI treatment. Immune-related adverse events encompass dermatologic/mucosal, gastrointestinal, hepatic, endocrine, and pulmonary toxicities. Other less common but important immune-mediated toxicities include rheumatologic, cardiovascular, hematologic, ocular, neurologic, and renal manifestations.

The management of immune-mediated adverse events depends on the nature and severity of the toxicity and has been discussed elsewhere in more detail [145,146]. Treatment of higher-grade toxicities usually involves immunosuppression with glucocorticoids. An escalation of therapy may include tumor necrosis factor-alpha antagonists, mycophenolate mofetil, or other immunosuppressive agents. Depending on the specific toxicity and grade, moderate and severe immune-related adverse events may require interruption of the checkpoint inhibitor and close monitoring while glucocorticoid immunosuppression is introduced. In such cases, the ICI should not be resumed until toxicities are down to grade 1 or less. For severe or life-threatening toxicities, a permanent discontinuation of checkpoint inhibitor therapy is usually indicated along with immunosuppression.

## 11. Conclusions

Even though the use of ICI in some gynecological cancers such as endometrial cancer has been promising, a better understanding of cellular and molecular parameters guiding response rates and survival in patients will be paramount to the optimization of future combination therapy regimens for the improved management or cure of these malignancies. To date, a myriad of combination clinical trials are in progress investigating the response of gynecologic cancers to treatment blocking PD-1 ligation. Endometrial cancer is encouragingly responsive to ICI therapy. Indeed, achieving a response rate of 57.1% with single therapy anti-PD-1 antibody (pembrolizumab) for EC strongly suggests that agents blocking the PD-1 axis may be useful and strategic alternatives for first-line chemotherapy failures. However, in contrast, advanced disease ovarian cancer remains a puzzling and difficult disease to treat with conventional or novel therapies, such that with ICI, there are response rates of only about 10–15% with monotherapy administration. Globally, approximately 295,414 cases of ovarian cancer are diagnosed annually, with about 184,799 deaths due to this disease each year. With these troubling statistics, in the near future, we hope that the outcome of the current phase of ongoing trials with unique combinations will unravel new combination therapy directions with ICI and other therapy agents, which are effective for the management of ovarian cancer especially, thereby providing much needed treatment options for this devastating illness.

## Figures and Tables

**Table 1:** Estimated new cases of female gynecologic cancer diagnoses and estimated deaths in the U.S. in 2020.

Organ	New Diagnosis	Deaths	^1^ (Deaths/New Diagnosis) × 100%
Ovary	21,750	13,940	64.1
Uterine corpus (endometrial)	65,620	12,590	19.2
Uterine cervix (cervical)	13,800	4290	31.1
Vulva	6120	1350	22.1
Vagina and others	6230	1450	23.3

^1^ The table shows the relative percentage of deaths for female gynecologic cancers based on the numbers of these cancers newly diagnosed. The uterine cervix is classified as cervical cancer, and the uterine corpus is classified as endometrial cancer (adapted from Siegel et al., 2020) [1].

**Table 2:** Clinical data for select immune checkpoint inhibitors evaluated as monotherapy in endometrial cancer.

Treatment ^1^	Study Phase	Endometrial Cancer Study Population	ORR	Reference
Anti-PD-L1 antibody				
Atezolizumab	Phase Ia	*n* = 15 Advanced or recurrent EC	Entire cohort—13%	Fleming [67]
Avelumab	Phase II	*n* = 31 Advanced or metastatic EC	MMRd tumors—26.7% MMRp tumors—6.25%	Konstanti-nopoulos [68]
Durvalumab	Phase II	*n* = 71 Advanced EC	MMRd tumors—40% MMRp tumors—3%	Antill [69]
Anti-PD-1 antibody				
Dostarlimab	Phase I/II	*n* = 110 Advanced or recurrent EC	MSI-H tumors—48.8% MSS tumors—20.3%	Oaknin [70]
Nivolumab	Phase II	*n* = 23 Advanced or recurrent EC	Entire cohort—23%	Hasegawa [71]

^1^ The clinical efficacy for multiple monoclonal antibodies targeting the PD-1/PD-L1 axis has been investigated in phase I/II trials, enrolling patients with advanced or recurrent EC. Abbreviations: EC, endometrial carcinoma; ORR, objective response rate; MSI-H, microsatellite instability-high; MSS, microsatellite stability; MMRd, mismatch repair deficient; MMRp, mismatch repair proficient.

**Table 3:** Clinical trials data for select immune checkpoint inhibitors evaluated as monotherapy in ovarian cancer.

Treatment ^1^	Study Phase	Ovarian Cancer Study Population	ORR	Reference
Anti-PD-L1 antibody				
Atezolizumab	Phase I	*n* = 12 Advanced or recurrent OC	Entire cohort—22.2%	Liu [121]
Avelumab	Phase Ib	*n* = 125 Advanced or metastatic OC	Entire cohort—9.6%	Disis [86]
Anti-PD-1 antibody				
Nivolumab	Phase II	*n* = 20 Platinum-resistant OC	Entire cohort—15%	Hamanishi [119]
Pembrolizumab	Phase II	*n* = 376 Advanced, recurrent OC Cohort A (*n* = 285) PFI/TFI of ≥3 to 12 months and less heavily pre-treated Cohort B (*n* = 91) PFI/TFI of ≥3 months and 3–5 lines of prior therapy	Cohort A = 8.1% Cohort B = 9.9%	Matulonis [120]

^1^ The treatment efficacy for multiple monoclonal antibodies targeting the PD-1/PD-L1 axis has been described in patients with advanced or recurrent OC. Abbreviations: OC, ovarian carcinoma; ORR, objective response rate; PFI, platinum-free interval; TFI, treatment-free interval.
